# Toward better AFIS practice and process in the forensic fingerprint environment

**DOI:** 10.1016/j.fsisyn.2023.100336

**Published:** 2023-06-03

**Authors:** Caroline Gibb, John Riemen

**Affiliations:** a University of Twente, Enschede, the Netherlands; b The Netherlands Forensic Institute, The Hague, the Netherlands; c National Police of the Netherlands, Manager National Criminal ABIS, the Netherlands

## Abstract

Automated Fingerprint Identification Systems (AFIS) are used in forensic operational environments worldwide. Traditionally, these systems are search systems only and have limited workflow to support forensic processes in recommended best-practice approaches. This paper addresses this issue by presenting best practice approaches and processes for AFIS in forensic environments. The discussion is divided into three parts. First, we identify three factors that can impact the performance of AFIS in forensic environments. Second, we discuss the search process and highlight how the National Police of the Netherlands (NPN) strategies to mitigate bias and error in their AFIS workflow. Finally, we briefly discuss other considerations for establishing best-practice. We offer resources for biometric managers and users, providing an overview of the best AFIS practices in forensic environments. We propose the NPN system as a benchmark and aim to foster discussions among AFIS communities to enhance AFIS practices and processes in forensic fingerprint environments.

## 1 Introduction

The Automated Fingerprint Identification System (AFIS) is a biometric technology designed to store digital representations of friction ridge skin (i.e., fingerprint, palmprint, footprint data) and rapidly search the database to establish a link between two impressions. AFIS provides an electronic database making it easier to maintain accurate records and access relevant information quickly [1]. AFIS can search through millions of fingerprints in seconds, enabling large-scale searching and automated recognition of possible suspects. It is primarily used for establishing the identity of individuals (border control, visa application), and for associating an individual with a mark in relation to a crime or public inquiry [2].

This system requires less human interaction, allowing for faster and more accurate results [3]. Nowadays, the enrolment of individuals can be fully digital (Live Scan), and biometric feature extraction or encoding can be fully automated. Additionally, it allows for the sharing of fingerprint data between different agencies and jurisdictions, improving collaboration and increasing the chances of identifying unknown individuals and solving crimes [2]. AFIS has increased speed and accuracy, reducing the time and effort required to process and examine fingerprint evidence, making it an ideal investigative tool in forensic-fingerprint environments.

### 1.1 AFIS workflow: from crime scene to search results

**Fig. 1:**
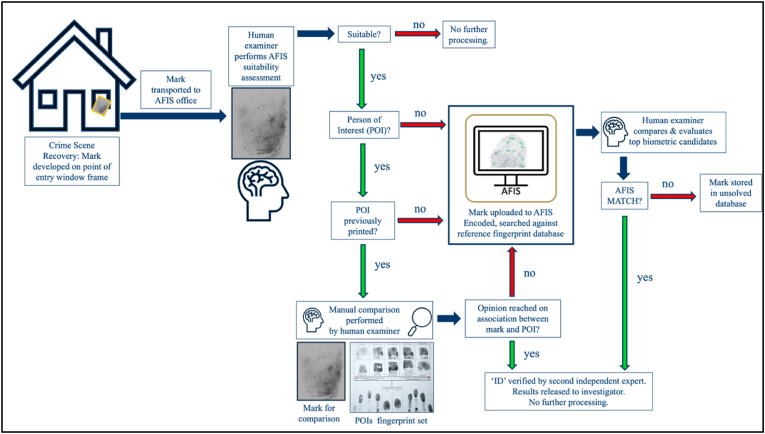
AFIS Workflow: From crime scene recovery to AFIS search results.

The general workflow of AFIS begins with the recovery of a mark from a crime scene, which can take the form of a digital file, a lift, or a photo. The mark undergoes a suitability assessment by an examiner and if there is a Person of Interest (POI) and this individual has been previously fingerprinted, the set of fingerprints can be manually compared by the examiner before the AFIS search. If during the manual comparison, the POI is identified as the source of the mark, the mark will be verified by another examiner, and an AFIS search is not necessary. If the POI is excluded by the examiner or an opinion cannot be reached, the mark is uploaded to the AFIS and a search is launched. The AFIS workflow, from crime-scene recovery to AFIS search results, is shown in Fig. 1.

### 1.2 The process of searching

Marks recovered from crime scenes are transferred to an operational fingerprint office for an AFIS search, either physically in the form of a lift or digitally in the form of an image. Prior to searching the mark, an examiner performs an initial assessment to determine if it meets the agency's requirements for search. AFIS suitability is dependent on agency policy, which may differ from the suitability criteria applied to other types of comparisons [4]. If the mark is deemed suitable, it is uploaded to the AFIS with relevant case information either through a digital scan or a digital file. Generally, the examiner orientates the mark in the upright position as it appears on the reference set. If the region of the mark is obvious because of ridge flow and other tell-tale signs (e.g., left thumb or right thenar area of the palm), the examiner can nominate the specific region for the system to search against. However, a search can also be launched unoriented without a specific finger or palm nomination. Once the enrolment process is complete, the mark is encoded.

The encoding of features is an essential part of a successful search in AFIS and involves the extraction of features, such as ridge endings and bifurcations. Feature encoding can be performed manually or automatically, or in combination. These encoded features create a map based on the geometric relationships and spatial frequency between each minutia [5]. The encoded minutiae-based feature set allows the system to search the database for a similar map or potential source [5]. NIST's ELFT-EFS test revealed that auto-encoding is as effective as manual encoding by trained examiners. However, a complementary effect was observed between auto-encoding and manual encoding, suggesting that a combination of both approaches may be ideal for AFIS performance [6].

Auto-encoding is a function performed by AFIS that involves automated extraction and annotation of features. This function is used during the enrolment of an individual's biometric reference set onto AFIS and is usually fully automated (“lights-out” 10-print fingerprint processing), requiring minimal, if any, human intervention [7]. Auto-encoding is very fast, and a single fully rolled fingerprint can contain between 40 and 100 minutiae [8]. A complete reference set of fingerprints comprises ten rolled fingers, including plain (slap) impressions, phalanges, and palms. Manually encoding an entire set of fingerprints is a human-intensive task, making auto encoding a desirable function from a resource perspective. The same holds true when processing a mark to search a database. When a mark has high clarity and requires minimal human intervention, auto-encoding can support faster TATs. Regardless of how the encoding is performed, once complete, the encoded mark is launched for search and compared to the biometric reference database.

From this reference database, AFIS generates a list of biometric candidates for comparison based on the similarity between the mark and the reference print. The search and subsequent comparison decision are categorised as either a hit or no hit. This can also be fully automated (e.g., lights-out scenario), but in most cases, the examiner will intervene to manually compare the top candidates, typically the top 10 to 20, and reach a decision on the result of the search based on their comparison and evaluation of the candidate list [7].

Biometric recognition, or a positive comparison decision implies that the mark and print are from the same source [9]. Non-recognition by the AFIS, or negative comparison decision, implies that the mark and print are not from the same source [9]. There are several reasons why a mark may not be recognised by the database, the most obvious being that the fingerprint reference set of the individual who potentially left the mark has never been fingerprinted. However, there are many other reasons why a search may be unsuccessful even when the database has the respective fingerprint set on the system. Several contributing factors can affect the performance of AFIS, which may inadvertently lead to errors, whether it is human, system, or a combination of both.

When a search produces a negative comparison decision, examiners can refine the search by conducting additional searches. To increase the chances of a positive result, examiners often duplicate and encode the mark in various ways. This approach is particularly useful when the reference set is of poor quality due to factors like damaged skin during enrolment or occupational markings such as bandaged fingers, fresh burns, or superficial cuts. For instance, refining the number of features to target specific areas of a large palm mark can generate multiple maps for the system to search against. Maximising AFIS strategies, that is, optimising the use of the AFIS system to achieve the best possible outcomes is especially vital in high-profile cases that pose a threat to public safety, such as terrorist attacks that usually involve unknown perpetrators. Although automating the process completely is desirable, human resources may still be necessary, depending on the offence (high profile), threat level to public safety, agency policy, and search approach.

## 2 AFIS performance in forensic fingerprint operational environments

**Fig. 2:**
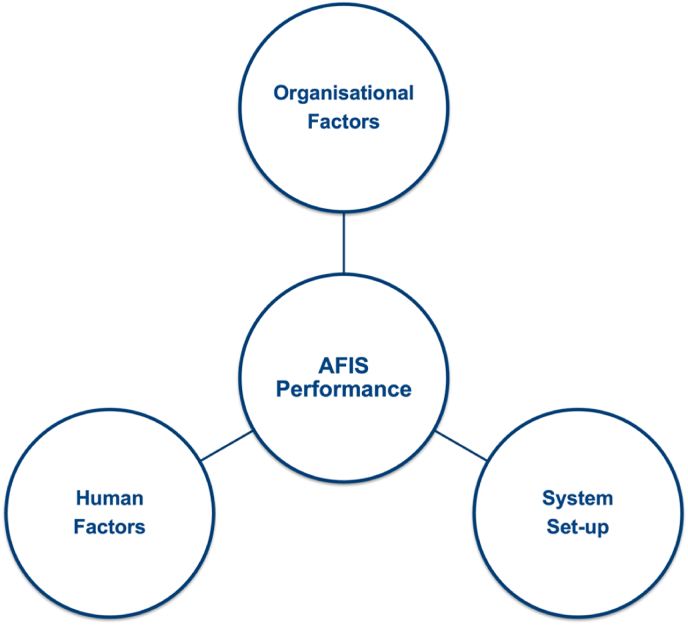
AFIS performance in a fingerprint operational environment is affected by three major influencing factors: (1) organisational factors such as safety and security, accessibility to information, and police hierarchy; (2) system setup, including the algorithm, workflow processes, and workstation setup; and (3) human factors such as workplace stress and well-being.

In a forensic operational environment, organisational factors can influence system capabilities and performance outcomes. Furthermore, the prevention of bias and error depends on the awareness of risk and opportunity, and the effective management of a combination of organisational factors, human factors, and the system setup. We suggest that these three factors have a major influence on AFIS performance within an operational forensic fingerprint environment (Fig. 2).

### 2.1 Organisational factors

#### 2.1.1 Public safety & security

The primary goal of police and their affiliated forensic service providers is to support public safety. Often, the AFIS is purchased, managed, and maintained by a police organisation, which has a clear strategy to ensure the safety and security of the public. For example, the Netherlands Police employs approximately 60,000 personnel responsible for primary policing tasks, such as crime prevention, crime investigation, public order maintenance, and assistance to citizens in need of help [10]. The investigation of crime has a clear goal: to solve the crime, identify those responsible, and seek justice through appropriate prosecutorial pathways.

#### 2.1.2 Operational hierarchy

In police organisations, a strong hierarchy exists among the ranks, where seniority and experience play significant roles in inter-human relations. Directives typically flow from higher-ranking officers, such as directors outside the fingerprint operational environment, to operational managers. When a forensic fingerprint department is affiliated with a police organisation, there is often pressured to reduce TATs to solve crimes faster. However, there is always a tradeoff. Directives to process a high volume of cases per day can lead to a decrease in the quality of the search. Furthermore, within the fingerprint operational environment, the hierarchy between examiners can have implications for the quality and accuracy of their work. (See 2.3.1. Hierarchy between examiners). In addition, close connectivity with the investigator can motivate examiners introducing motivational bias. This means that the examiner may have a desire to reach a positive comparison decision, either to help police informants or to be recognised as the examiner or department that solved the case and got the “bad guy” off the streets. By implementing appropriate case management strategies, such training and quality control measures, agencies can work toward minimising the influence hierarchy and motivational bias.

#### 2.1.3 Managing task-irrelevant information to reduce bias in AFIS search

How much information do you need to perform the task of searching? Fingerprint examiners play a key role in criminal investigations, but their ability to perform tasks effectively can be influenced by task-irrelevant information. This is particularly applicable in police organisations where examiners may have a dual function: processing the crime scene for marks and subsequently searching for the marks they recover. This is not to say that dual functions are bad, unreliable, or dishonest [11]. Rather, exposure to investigative information can introduce risks, as initial sources of information may drive crime scene investigation and recovery efforts and often include very specific requests from investigators on what surfaces and exhibits they would like to examine for marks [12].

Task-relevant information includes knowledge of the item, the location of the mark on that item, the development method used to visualise the mark, and the type of surface from which the mark was recovered. This information is necessary for the examiner to effectively perform the task. However, when task-irrelevant information, such as knowledge about the suspect's criminal history or alibi, is introduced, it can influence the examiner's judgment or opinion relating to a case without conscious awareness [13].

To prevent bias from task-irrelevant information, an effective context management system is necessary to remove such information and prevent cross-contamination prior to an AFIS search [14]. This system can protect the scientific integrity of the evidence and improve the quality of search results. Fingerprint examiners should be trained to effectively manage task-irrelevant information and focus on the relevant information required for the task. Thus, examiners can reduce the risk of bias and error in the AFIS search process, leading to more accurate and reliable results.

### 2.2 System set-up

#### 2.2.1 The algorithm

The accuracy of the algorithm is critical to the performance of the system and outcomes of the search. AFIS managers are encouraged to evaluate the technical performance of their biometric systems [15]. In addition, the performance of AFIS is regularly evaluated using international benchmarks. The most accurate submission in the latest evaluation achieved a false negative identification error rate, a FNIR^4^ of 1.97% for the left index finger and 1.9% for the right index finger when compared against an enrolment set of 100,000 subjects (1 million fingerprints): a false positive identification error rate, a FPIR^5^ of 10^−3^ [2] Additionally, the NIST ELFT program is specially designed to evaluate the accuracy of AFIS technology in the forensic operational setting making it an ideal benchmark for the AFIS [7].

AFIS managers can also benchmark the AFIS by providing them with recommended best practices and international standards [15]. The NPN chose to organise a benchmark in collaboration with the University of Twente as part of the tender process. The benchmark outcome played a significant role in the selection of the provider for the AFIS solution. The NPN AFIS strategy focuses on achieving maximum accuracy of the system from a business standpoint. Consider a difference of 3% in the AFIS accuracy. In this scenario, if the agency processes 10,000 marks per year, this is the difference between the system accurately recognising 300 individuals. If, among these 300 ‘missed’ individuals, five were connected to two homicides, the cost of public safety would be significant. In addition to the loss of human life, one study estimated that one homicide costs society around $17.25 million [16].

Considering the role that the AFIS can play in early intervention in preventing the potential escalation of criminal activity, investing in the accuracy and performance of an AFIS, and the life cycle of the AFIS (6–10 years), is significant. It is essential to understand the potential limitations of the purchased algorithm and have a strategy to mitigate these risks through the entire chain. While accuracy is important, other factors to consider include accuracy, speed, cost, and ease of maintenance (Section 7.3).

#### 2.2.2 Workflow processes

Effective workflow processes are vital for the successful use of AFIS. This includes the operational and performance requirements of the system as well as the policies surrounding case handling before and after AFIS processing. In accredited laboratories, international standards such as ISO 17020 and 17,025 provide a framework for regulating systems and processes to ensure accuracy and reliability. System specifications such as the management of contextual information, blind approaches, and court charting can positively enhance the AFIS operational environment through error mitigation.

#### 2.2.3 Workstation set-up

The workstation setup of examiners is critical for their interaction with the AFIS system [17]. Proper ergonomic workstation setups (height adjustable desks), suitable environmental lighting, and screen size and resolution are important considerations to prevent injuries to enable better performance [18,19]. Operational managers must be aware of the impact that even one full-time examiner on sick leave can have on the daily functioning of the forensic operational environment. To further optimise performance, managers should strive to create a distraction-free environment. Factors such as lighting and noise levels and other distractions should be evaluated and managed [18]. Implementing measures such as applying foil on windows to block sunlight from shining on screens and using headsets, or other noise cancelling devices, to reduce noise and distraction may be a necessary solution. Additionally, having a designated front office to answer calls, rather than having employees answer them at their desks is also another effective strategy to reduce interruption during AFIS processing.

#### 2.2.4 Screen resolution and quality

The choice of display holds significant importance for examiners as it plays a crucial role in supporting their interaction with the AFIS, particularly in terms of screen quality and resolution.

Additionally, the AFIS workstation display is a critical tool for two primary reasons. Firstly, it promotes workplace health and safety by preventing eye strain [20]. Secondly, it plays a vital role in ensuring accuracy by providing high-quality and clear representations of the intricate details of fiction ridge required for comparisons.

The level of friction ridge detail displayed in an image depends directly on the monitor's resolution and other technical specifications, such as self-calibration, luminance, colour contrast ratio, and physical size [21]. Selecting high-quality displays with sufficient resolution is essential for accurately and optimally displaying friction ridge detail (prints and marks). It is important to note that various types of displays are available, ranging from inexpensive consumer-grade desktop displays to more expensive medical-grade displays used in digital pathology [21]. While cost is a factor during procurement, the chosen display must align with the intended task. Therefore, understanding the examiner's specific needs and prioritising their support is critical when deciding on the appropriate display to invest in.

Meeting the minimum standards for image size and screen resolution is vital to ensure the production of high-quality images that support all phases of identification [1]. Even if the AFIS workflow does not directly contribute to decision-making, a monitor is still used for carrying out the ACE-V process. Hence, careful attention must be given to selecting and maintaining the display. Based on the experience of the NPN, both AFIS vendors and users often lack awareness of the associated risks and potential benefits. Nevertheless, it remains crucial to choose a monitor that suits the intended purpose, offering sufficient resolution and contrast to accurately display high quality friction ridge images on the screen, whether in full-screen or split-screen mode [22].

AFIS managers should develop a maintenance strategy and consider the lifecycle of their screens. In the event of a screen breakdown, it is important to assess if the exact same screen is still available or if it would be replaced by a newer version that may not necessarily be better. Considerations should be made regarding reliance on the vendor or the internal IT department and their willingness to provide a screen that is fit for the intended purpose, rather than a standard consumer-grade desktop display. Additionally, for transparency, reproducibility, auditability, and explainability of the fingerprint examination process in court, it is important to utilise computer-supported processes with software that audits and stores every step.

### 2.3 Human factors

Even in a computer-assisted and largely automated environment, bias and errors can still occur. Numerous studies have examined the effects of human factors on reasoning, which go beyond context and confirmation bias, and include workplace stress and well-being [23], fatigue, policy, personality, training, and knowledge [[24], [25], [26], [27]]. Error is not a result of ethical issues, bad apples, or examiner immunity [28]. Error extends to factors such as hierarchy between examiners, passing over task-irrelevant information, human error by non-recognition (misses), and the increased likelihood of exposure to Close Non-Matches (CNMs) owing to increasing biometric databases.

#### 2.3.1 Hierarchy between examiners

The ‘best’ examiner is often correlated with the number of years of experience in the field. However, senior and experienced examiners can also influence the judgment of other examiners. Research has demonstrated that years of experience does not necessarily lead to greater examiner performance [14]. In an operational environment, the first examiner reaches their conclusion and hands over their results to the second (often a more senior) examiner for verification. Are you bold enough to disagree with the opinion of highly respected senior examiner? Is there a possibility of overruling? If you observe a difference in the corresponding minutiae, will you be made a fool expressing your opinion? Verification is a good measure to minimise risk, but the details include how the information is passed over from one examiner to another.

#### 2.3.2 Passing over information

In the organisational factors section, we briefly discussed the police hierarchy, their mission towards public safety, and accessibility to information. In some police organisations, measures are taken to minimise access to information. A fingerprint examiner should ideally be able to work pressure-free with information on a need-to-know basis. It is important to consider which information is shared and which information is not needed to perform the function [18]. Perhaps the first examiner offered an unsolicited opinion to the second examiner when passing the case for verification. For example, “here is an easy identification, not complex you will see”. Alternatively, the first examiner may offer observations, which could influence the second examiner's interpretation. For example, “there is a nice configuration of features to the left of the delta but it's a little distorted, I'll show you.” Other influencing scenarios relate to the release of the results, e.g., “I promised the investigating officer I would give them a result before lunch,” or “the investigating officer needs a name ASAP.” The pressures imposed on the verifier influence their thresholds and opinions [29]. To prevent this, effective case management systems should be implemented to remove task-irrelevant information at the front end. Additionally, strategies such as creating AFIS work silos and separating tasks are also effective (Section 6.4).

#### 2.3.3 Human error by non-recognition (false exclusion)

Several studies have shown that the rate of false exclusions far exceeds that of false identifications [30]. The black box study by FBI/NOBLIS reported a false-positive rate of 0.1% and a false-negative rate of 7.5% [31]. NPN has seen similar rates in operation. However, the error rates for palms, particularly erroneous exclusions, have been shown to be much higher [32]. Missing the correct donor by an examiner can have different causes, such as loss of concentration at the end of the day, a colleague asking for something during verification, or a mark with a different orientation than the presented finger to verify. Another influencing factor is the biometric candidate list. The position on the list and the biometric score can also influence judgments [29]. If the algorithm is accurate and 95% of the hits are found on rank 1, the risk is that examiners will pay less attention or even skip the lower biometric candidates.

#### 2.3.4 Close Non-Matches (CNMs)

A Close Non-Match (CNM) refers to two friction-ridge impressions that share similar features, making it difficult to distinguish the two impressions [33]. An increasing number of biometric databases contribute to an increased likelihood of shared coincidental similarity between the two impressions [34,35]. There can be a high level of correspondence between the questioned mark and the suggested biometric candidate on the AFIS, leading to potential errors. In this scenario, the potential for error increases because of the complexity of the comparison and the close resemblance of the features.

This emphasises the importance of providing examiners with training to recognise high-risk scenarios and to offer them guidance on how to best document and evaluate these situations [36]. Furthermore, training opportunities for examiners working under difficult conditions could include the use of statistical tools to inform judgments and help express the degree of association when source-level identification or exclusion cannot be determined. While some agencies may opt for insufficient and inconclusive opinions, providing examiners with statistical tools to predict [37,38], and quantify the weight of an association could add value to their opinions [6,39].

Despite ongoing research into evidence-based strategies for dealing with CNMs [40,41], the risks are evident, even without comprehensive data. The larger the size of the database, the greater is the likelihood of encountering a CNM in the candidate list. Relying solely on experience is not sufficient to safeguard examiners [42].

## 3 Understanding AFIS limitations

“Humans face limitations that machines do not, but machines also face limitations that humans do not.” [43].

### 3.1 Reproducibility of features

For AFIS to produce a result, the questioned mark must exhibit features that are reproducible to effectively search the reference database [5]. However, latent (invisible) marks recovered from crime scenes are always partial recordings of the friction ridge skin, and because of the mechanism of touch (pressure and movement), they can often be of poor quality, exhibiting background noise from a variety of distortionary factors (e.g., deposition pressure, surface, and development technique). These factors directly affect the reproducibility and reliability of features. Reproducibility of features upon deposit of the friction ridge skin with a surface is the most important factor to enable a comparison and establish the degree of similarity between two impressions.

### 3.2 Auto-encoding

**Fig. 3:**
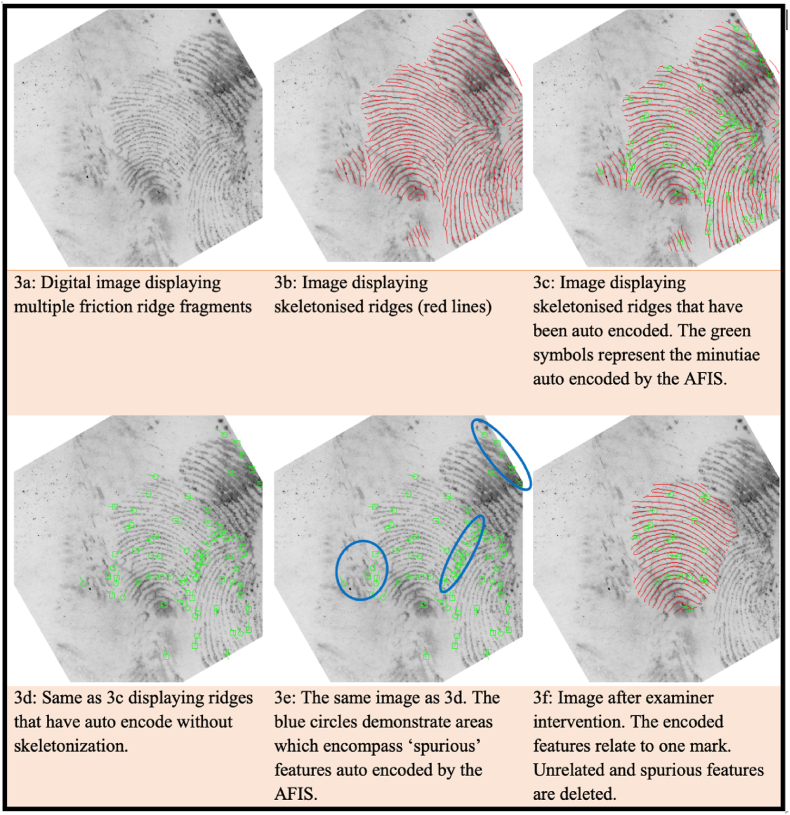
Series of images and descriptions demonstrating the uploaded mark before encoding is performed (3a), followed by the mark displaying the skeletonised ridges (red lines) (3b). The following images demonstrate how the system encodes multiple impressions in the absence of examiner intervention (3c, 3d, and 3e). The last image (3f) demonstrates the mark after spurious features have been manually removed by the examiner. The mark is now ready for search. If other marks are deemed suitable for the AFIS search, they will go through the same process as described. (For interpretation of the references to colour in this figure legend, the reader is referred to the Web version of this article.)

When an individual's set of fingerprints is taken for identification purposes (e.g., live scan), feature encoding is performed automatically, and the system may extract noise or spurious features that do not represent actual features (Fig. 3). This could be because of the condition of the individual's hands (occupational markings), or a result of deposition effects such as finger slippage during live scan capture. Additionally, features such as incipient ridges may be encoded as features by the system but not by humans. Naturally, incipient ridges are features, and if reproducible and reliable, they will be used during the comparison. Auto-encoding is sometimes a more reliable approach from the AFIS perspective because these features and other anomalies (e.g., scars) are encoded during the enrolment of the fingerprint set. If these features and anomalies are captured during enrolment and are permanent or present on the individual's skin during contact (e.g., scars), they will also be present upon the development and visualisation of the mark. Although this is a notable limitation, these non-traditional features should be considered by the examiner when encoding a mark. As discussed later, the auto-encode function should be used first to ensure the best representation of the biometric features, even if they fall outside the traditional definitions and approaches to manual feature encoding. The examiner will intervene if the first search is not successful and re-launch the mark after manual addition or removal of minutiae. This is important if the reference set is of poor quality and has missed capturing areas during enrolment. While accuracy is undoubtedly the most important factor in an AFIS, and auto-encoding can achieve this, human resources are also essential.

### 3.3 Searching and manual encoding

In situations where there are multiple marks located closely together on an item, and it is not possible to photograph or lift them separately, examiners must separate each mark and clearly indicate to the AFIS the precise area to encode. This is necessary because not all systems can differentiate between each mark and will instead encode all available information. Separating marks can be carried out using the lasso function to define a region of interest and indicate to the system which area requires auto-encoding. Other tools can be used to assist the examiner in identifying spurious features for elimination, for example, the skeletonise tool can be used to decrease the background noise of the ridges and assist the examiner in locating areas of unnaturally interrupted ridge flow. Fig. 3 below demonstrates how tools like the skeletonise function can be used to assist the examiner in identifying areas that have been encoded by the system that require human intervention and manual clean-up and deletion of spurious features. Manual encoding is highly recommended in cases where the quality of the mark is so poor that human expertise exceeds the system's ability to extract and encode features accurately.

## 4 Take a good approach

“The sole objective of the investigation of an accident or incident shall be the prevention of accidents and incidents. It is not the purpose of this activity to apportion blame or liability.” [44].

Perhaps the most significant difference between the forensic fingerprint field and other high-stakes decision-making domains, such as aviation, is the cultural attitude toward error prevention and management. In forensic science, there is a ‘blame culture’ that holds individuals accountable for mistakes that occur [18]. Historically, if examiners made an error and were exposed, they could have their certification revoked or, in extreme cases, fired. This can create a culture of fear and reluctance in reporting errors. However, in fields such as aviation, a healthy approach to error prevention has been adopted. The final aviation investigation report focuses on promoting safety and does not assign blame or liability to individuals involved in accidents [45]. This has led to a more open and transparent culture, in which errors are reported and addressed openly, with a focus on improving safety and preventing future accidents.

One way that the forensic field could adopt a similar approach is by establishing initiatives like the “CrashProof Lab”, or CrashProof Knowledge Centre in aviation [46]. This concept aims to investigate crashworthiness aspects of unconvential aircrafts using state-of-the-art modelling techniques, validated models, testing methods, and testing hardware to reduce errors and improve safety [46]. In the forensic field, similar initiatives could be established, such as an “Error Proof Lab,” aimed specifically at reducing human error across feature-comparison disciplines.

The primary objective of such initiatives in the fingerprint domain is to push the examiner and AFIS beyond the current levels of performance. This could involve developing relevant forensic tasks that incorporate CNMs to promote errors, followed by a review of how errors occurred [47]. By studying the underlying factors that contribute to errors [36], researchers could develop new tools and techniques to prevent them from occurring in the first place. Importantly, the lab can serve as a testing ground for examiners to test, train, and trial new tools and technologies. By rigorously testing these innovations in a controlled environment, both researchers and examiners can better understand their potential impact on real-world scenarios.

Adopting a more proactive approach for error prevention in forensic science could lead to a more open and transparent culture. By focusing on promoting safer justice outcomes and improving performance rather than assigning blame, forensic science can move toward a more collaborative and productive future. Nevertheless, in the absence of such initiatives, strategies to safeguard examiners from bias and errors are critical in forensic fingerprint environments.

## 5 Safeguarding forensic fingerprint examiners against human factors

“Errors can be prevented by designing systems that make it hard for people to do the wrong thing and easy for people to do the right thing.” [18].

Errors in fingerprint examinations can have serious consequences, emphasising the need for safeguards against errors. Public exposure to errors and the resulting recommendations have highlighted the need for safeguards in the fingerprint domain [11,18,35,48,49]. The FBI misidentification in the Brandon Mayfield case in 2004 was the first AFIS-related error to be reported on a global scale. The subsequent review of the error revealed several contributing factors, including confirmation bias and “unusual similarity between certain friction ridge details.” These findings have led to the publication of several recommendations aimed at improving the methodology of friction ridge examination and preventing future errors [35].

Published recommendations focus on preventing all errors and include ‘misses’ or false-negative conclusions. False negative conclusions can also significantly impact public safety and public trust in forensic science. Missing a potential candidate during a search can lead to a perpetrator committing more offences. Operational managers must also prioritise how to deal with misses, be it human error, system error, or a combination of both. Strategies to prevent missing the reference print in a search or candidate list are just as important as preventing false-positive conclusions. As forensic fingerprint examiners continue to play a critical role in forensic investigations, strategies are required to prevent bias and errors.

A debiasing strategy is any technique, tool, or method designed to eliminate bias and reduce its intensity and frequency [50]. Various known safeguards in the forensic operational environment include context management strategies [51], documentation strategies to capturing examiner reasoning, Linear Sequential Unmasking (LSU) strategy to support the examiner in completing the analysis prior to exposure to a reference print [28], and the application of a colour-coding system like GYRO (Green-Yellow-Red-Orange) [52] to indicate the level of confidence and examiner has in a feature.

Additionally, strategies aimed at reducing the variability involved in subjective human-based methods, such as consensus approaches, are also helpful [53]. Research is currently underway to explore automated approaches to reduce human elements, such as automated quality assessments and evaluative approaches [54,55] (D. A [6,56]. Promisingly, the discipline is beginning to explore automated lights-out latent processing in daily workflows [57].

## 6 The AFIS of the NPN

This section provides the AFIS community with effective debiasing strategies that have been successfully implemented by the NPN in their AFIS workflow. It is intended for AFIS managers and examiners who wish to learn about the system's capabilities and best practices to follow. The following strategies have been implemented to safeguard NPN examiners against bias and improve search effectiveness: Context management strategy, Confirmation bias strategy, first search auto encode: ACE-ACE Workflow, and Multiple procedure and blind verification strategy (consensus in practice). Below, we discuss these strategies in detail in the context of the NPN AFIS.

### 6.1 The NPN AFIS workflow: an overview

**Fig. 4:**
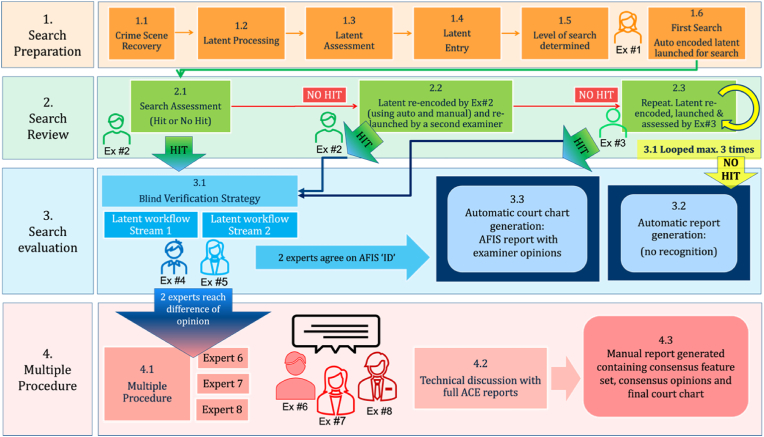
The NPN AFIS workflow can be divided into four stages, each of which is colour coded for clarity. Each examiner is depicted at each stage; the number (Ex #1, #2, and so on) represents another independent examiner, meaning that if a multiple procedure is reached, a total of eight independent examiners are involved in the entire process. The stage number is displayed on the far-left side to represent the processing stage. The four stages were as follows: (1) search preparation, (2) search review, (3) search evaluation, and (4) multiple procedures. The first three stages are performed on the AFIS, whereas stage four requires a separate more involved approach and involves three additional independent examiners to perform ACE and manually before coming together for a technical discussion. (For interpretation of the references to colour in this figure legend, the reader is referred to the Web version of this article.)

The NPN has established a standard workflow where AFIS processing is the first step, which can be followed by a more thorough multiple procedure (Fig. 4). The tasks are separated, and the number of examiners involved in the case depends on the ‘Level of Search’ assigned to the mark during registration.

**Fig. 5:**
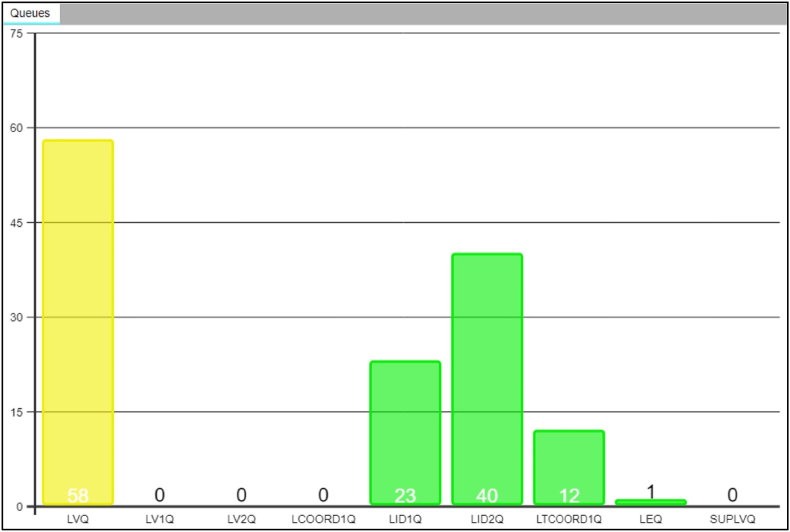
The NPN AFIS work silos. The coloured bars represent the number of marks in each silo. From left to right. The yellow bar labelled ‘LVQ’ indicates the number of marks in the queue for the first examiner to auto-encode and launch for search. The second and third green bars, labelled ‘LID1Q’ and ‘LID2Q,’ represent the number of marks (23 and 40 respectively) that require a full ACE examination. The third green bar, labelled ‘LTCOORD1Q’ (12), indicates cases with a difference of opinion (complex comparisons). The fourth green bar on the far-right side, labelled ‘LEQ’ (1), indicates the number of negative comparison decisions (no hits). (For interpretation of the references to colour in this figure legend, the reader is referred to the Web version of this article.)

The first examiner initiated in the process is only responsible for launching the auto-encoded mark for its initial search against the database and does not assess the result of the search (Figs. 4 and 1.6). Instead, the second examiner retrieves the mark from the AFIS workflow (Fig. 5) and performs the initial search assessment verifying if there is a hit or no hit. If the result is a hit, the mark enters a separate workflow, where a blind verification strategy is performed by two additional examiners who will conduct a full ACE on the mark. On the other hand, if the result is a no hit, the second examiner will re-encode the mark using a combination of auto-encoded and manually encoded features before relaunching it for search. If the mark remains a no hit, the third independent examiner will retrieve the mark from a work silo and repeat the process. If the mark is still a no hit the system generates an automatic no recognition report.

If the mark has hit with the updated encoding, it will enter a blind verification strategy, and two additional independent examiners will perform a full ACE on the mark. For this purpose, the mark automatically enters an “ID” silo, for a 1:1 comparison of the mark with a print. Two additional experts are initiated to perform ACE. Each examiner conducts an ACE independently on the mark, and if it is individualised, each examiner will manually chart out points for demonstration to the court in addition to their full ACE reports. On completion, the AFIS will automatically generate a court report with the two examiner opinions and their court charts. However, if the examiners reach a difference of opinion three additional independent experts are initiated, and multiple procedure is launched.

Prior to carrying out this complex procedure, the police forensic unit consults whether it is necessary to perform this labour-intensive task. In many cases, this step is deemed unnecessary because other fingerprint identifications have already been made on the same individual. If required, this procedure can always be conducted at a later stage, such as when requested during court proceedings. However, it is reported that the process wasn't initiated because of the consultation.

During the multiple procedure, ACE will be performed manually and captured digitally before the three experts come together for a technical discussion. During the technical discussion one of the examiners assumes the role of the technical chair. There is no hierarchy between the examiners, and the chair position is temporary. Each examiner presents their analysis, comparison, and evaluation to the group. Based on this discussion, a consensus on the most reliable minutiae is reached, and this consensus feature set is used for comparison and evaluation. If there is still a difference of opinion, the most conservative opinion prevails for the final consensus (i.e., inconclusive). The case file contains all the information, and the process is completely transparent. The NPN AFIS workflow is illustrated in Fig. 4.

The table below describes the NPN AFIS workflow, which is divided into four stages. The progression to Stage 4 Multiple Procedures generally involves a complex examination (poor-quality mark, reference print, or both). Most of the cases completed at the NPN are through the AFIS workflow.

**Figure:**
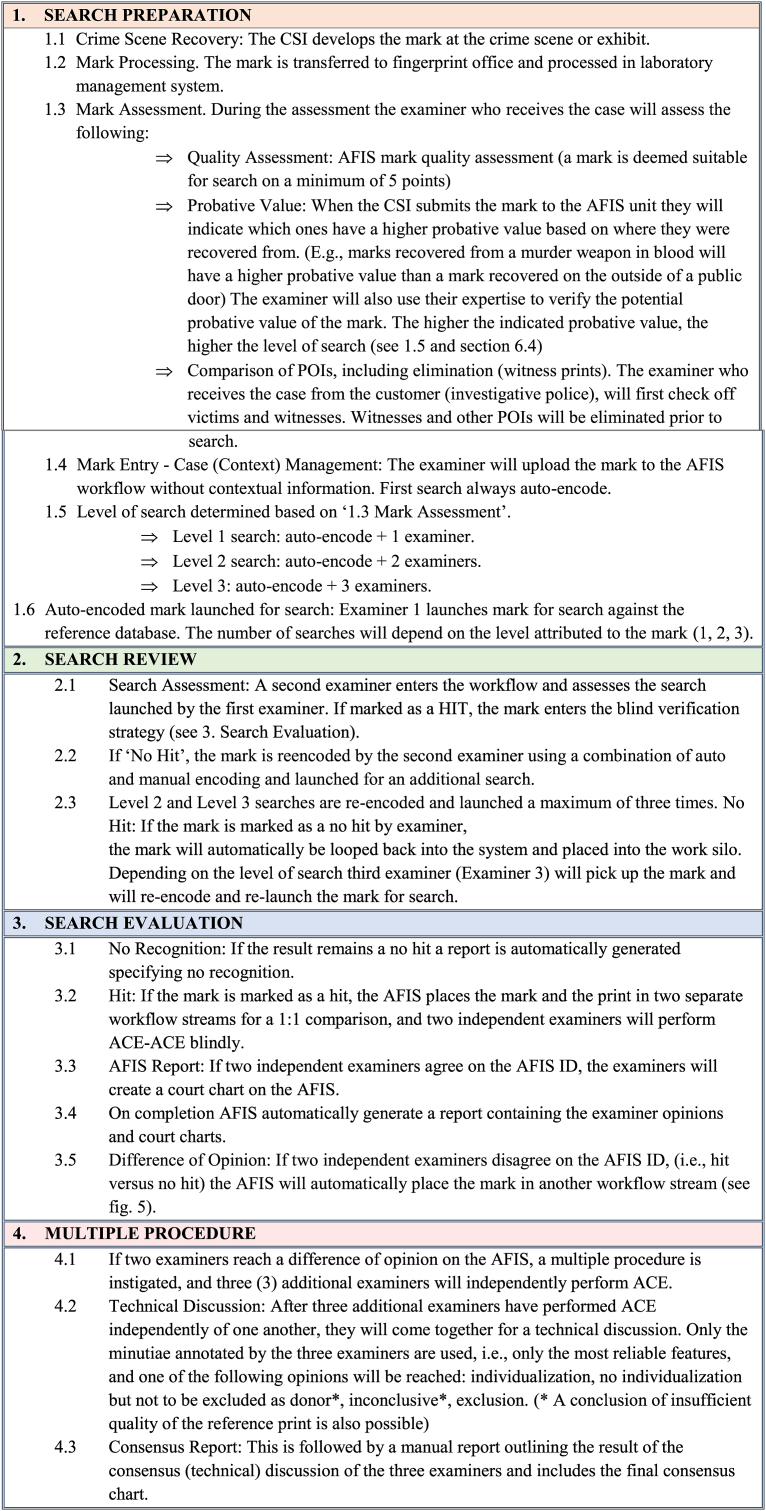

### 6.2 Context management strategy

The NPN process involves separating the case information from the AFIS process. The examiner responsible for assessing the quality of the mark and determining the level of search is the only examiner with knowledge of the case context. Once the mark is entered, the AFIS displays only minor case information, such as the police case number, unique mark identifier, and the unit that entered the mark. In most fingerprint departments, the level of examination effort is determined by the offence and mark quality, but at the NPN, the level of effort for every mark is based on its probative value. This value is established at the crime scene based on the location of the mark in relation to the committed crime. Consider the following circumstances: an assault was committed inside an apartment, and the offender exited through the front door down a hallway. The CSIs recovered two marks, one on the inside of the apartment door and the other, a mark in blood, outside of the apartment on the wall in the hallway. In this scenario, the mark in the blood outside the apartment will have a higher probative value than the mark inside the apartment door. As a result, more effort is put into the mark in the blood than on the front door. Furthermore, examiners were not permitted to examine cases in their operational regions. For example, if the mark was recovered in Amsterdam, examiners from other regions would process the case, and those from Amsterdam would not access it during processing. This effort is managed by an NPN central server.

### 6.3 Confirmation bias strategy

By the time an examiner retrieves a mark for analysis in the AFIS workflow, any task-irrelevant information is removed. The candidate list in the NPN AFIS only includes the reference print from the database, file number, and score, whereas names and other irrelevant information are inaccessible. To prevent circular reasoning, exposure to the reference print occurs only after the analysis is completed. This is particularly important for lower-quality marks, as there is a tendency to work backward from the better-quality reference print. This could lead an examiner to attempt to confirm features in the reference print that may not exist in the mark, which can in turn influence perception, especially in highly distorted areas and CNMs. As mentioned earlier, the biometric score can influence the examiner and bias the comparison (Section 2.3.3). To mitigate this, the NPN considers a strategy to remove the score and shuffle the biometric candidate list for comparison. Further research is needed to support this strategy and the extent to which it may affect the outcomes and operations of the examiner.

### 6.4 AFIS work silos and the separation of tasks

Cases are managed through the AFIS and organised into their respective silos, while examiner tasks are separated throughout the process. During ACE-ACE, the examiner cannot access the same case in the other stream (it is ‘greyed out’) and the examination is performed blindly. All the cases have only one AFIS case number and contained no other information about the case. Only after completing their analysis of the mark will the examiner be presented with the reference print nominated by the first examiner. The NPN AFIS work silos and the number of marks in each silo are shown in Fig. 5.

The first yellow bar labelled ‘LVQ,’ indicates the number of marks in the queue for the first examiner to auto-encode and launch for search (58). This first represents the system recognition step. The second and third green bars, labelled ‘LID1Q’ and ‘LID2Q,’ represent the number of marks (23 and 40 respectively) that require a full ACE examination after a positive comparison decision or hit has been indicated during the system recognition step. Next, a second examiner will compare the mark with the reference print and record their conclusion in the AFIS, along with the system comparison decision. Next, the mark re-enters the AFIS workflow and is placed in a second work silo to ensure that the third examiner is completely blinded to the previous examinations, knowledge, or information about the case. A third examiner will perform and fully document their ACE analysis, and the resulting conclusion will be recorded and stored in the AFIS. The third green bar labelled ‘LTCOORD1Q,’ (12) indicates a difference of opinion has been reached by two previous examiners and typically relates to a complex comparison. The fourth green bar on the far-right side, labelled ‘LEQ,’ (1) indicates the number of negative comparisons decisions, or no-hit. These marks are looped back into the system and re-encoded by another examiner. The number of loops depends on the level of search (1, 2, or 3) attributed to the mark during registration. Finally, a report is automatically generated and recorded in the AFIS to complete the process.

### 6.5 First search: auto-encode

**Fig. 6:**
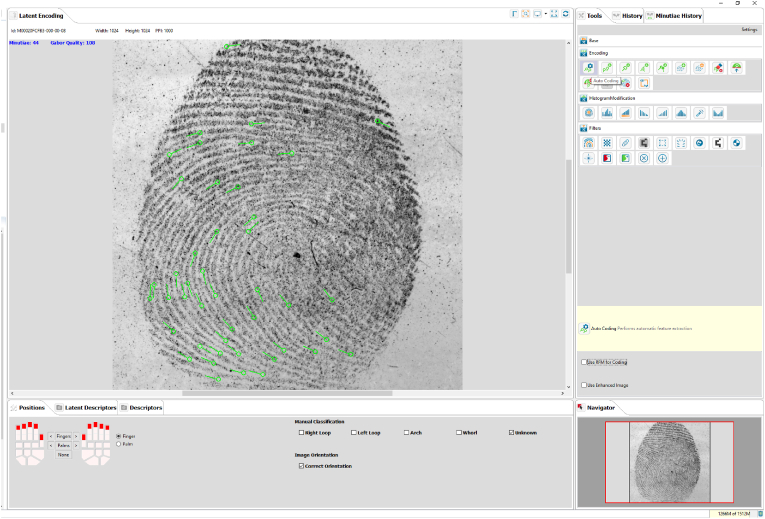
A Screenshot of the NPN AFIS interface, with the analysis screen on the left-hand side displaying the auto-encoded fingermark. The green symbols in the image indicate auto-encoded features. (For interpretation of the references to colour in this figure legend, the reader is referred to the Web version of this article.)

These days algorithms have become so advanced that they often require minimal human intervention (Singla et al., 2020). The most recent NIST Evaluation of Latent Friction Ridge Technology (ELFT) showed that the auto-encoding of marks is comparable to the manual encoding carried out by examiners with respect to the performance of AFIS [6]. This does not imply that the examiner and the system encode the same (see 3.2); however, with regard to performance, using the auto-encode tool for the first search is sufficient, followed by the examiner cleaning up the annotated mark if necessary. Prior to searching the database, the auto-encoding function is initiated by the examiner (Fig. 6). From an operational perspective, auto-encoding saves time, allowing for a better allocation of resources and expertise. It is efficient, easy, fast, and often requires only a single search. This reduces the workload and provides an opportunity for examiners in training to build up their experience while contributing to their professional development and expertise, as well as contributing to office productivity. Moreover, this frees up resources for more skilled examiners to focus their energy on more complex and difficult cases. Highly distorted marks exhibiting poor quality and clarity of features still pose challenges to both humans and systems. Instead, to obtain the most efficient results from the system, the first search should always be auto-encoded, followed by human intervention or a manual clean-up if necessary.

### 6.6 Approach to friction ridge examination (ACE-ACE workflow)

**Fig. 7:**
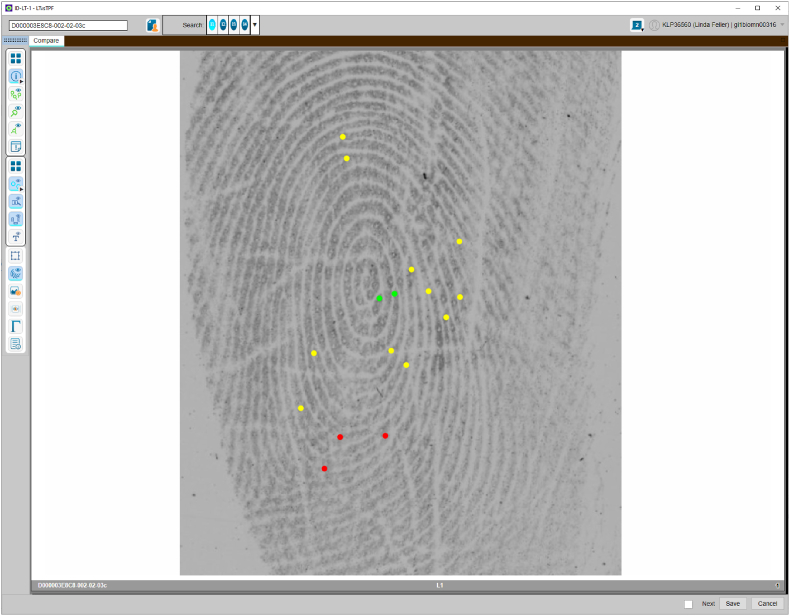
Screenshot of the NPN AFIS interface in analysis mode, displaying a mark annotated using (green-yellow-red) traffic light system approach. The GYR system was used to indicate the examiner's confidence level in the observed features. The green colour represents a high confidence in observing this feature in the same relative position, type, and shape if provided with the same source reference print. The yellow represents moderate confidence indicating that the examiner is confident a feature is present in but is unable to discern the exact type of feature due to distortion or connective ambiguity or a combination of both. Red features appear in distorted areas and represents low confidence in the observed feature. (For interpretation of the references to colour in this figure legend, the reader is referred to the Web version of this article.)

The NPN AFIS system employs a linear ACE-ACE workflow to minimise the transfer of irrelevant information between the examiners. Linear ACE-ACE (rather than ACE-V where the second examiner waits for the first examiner to complete their ACE) refers to a fully independent analysis, comparison, and evaluation performed by two experts. The examiners perform ACE blinded to other opinions (blind verification). Furthermore, the workflow incorporates an LSU approach whereby the analysis is performed first and prior to exposure to a reference print in combination with a colour-coding scheme (similar to GYRO) to capture examiner reasoning during independent examinations. For greater contrast during the comparison, the NPN uses blue (B) instead of orange (O) to indicate circular reasoning. Regardless of the colour used during the comparison, it is essential to highlight that features have been annotated after exposure to the reference print, from print to mark. For the remainder of this paper, we use the term GYR when discussing the features coded during the analysis phase, and GYR(B) for the comparison phase. To begin the analysis the examiner selects the mark at the top of the AFIS search queue and performs a full analysis of the mark using GYR. The following example demonstrates a mark annotated using GYR on the NPN AFIS interface (Fig. 7).

### 6.7 Circular reasoning strategy

**Fig. 8:**
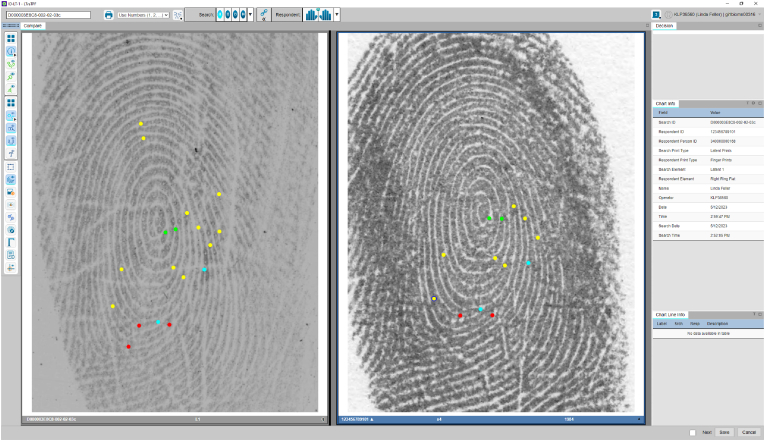
Screenshot of the NPN AFIS interface demonstrating the comparison screen using the NPN GYR(B) approach. Please note that this is solely for demonstration purposes to showcase the colour scheme, and not all features were annotated. On the left-hand side, you can see the mark, and the print is displayed on the right-hand side. The examiner is guided by the GYR features marked during the analysis. The blue features are used to represent minutiae that have been observed with the assistance of the reference print (circular reasoning). (For interpretation of the references to colour in this figure legend, the reader is referred to the Web version of this article.)

After the analysis has been completed and locked, the examiner can access the reference print that appears on the right side of the screen (as shown in Fig. 8) and a comparison will begin. The features annotated during the analysis using GYR will guide the comparison, and the examiner will search for the corresponding feature in the print. If the reference set aids in observing additional minutiae, it is marked in blue to indicate that circular reasoning has been used. The follow figure demonstrates the comparison screen on the NPN AFIS interface and displays features annotated using GYR(B) (Fig. 7).

### 6.8 Recording the conclusion: AFIS generated court charts

Forensic examinations must be transparent, reproducible, auditable, and explainable in court. To support this notion, the NPN chose to be completely transparent to minimise administrative work and reduce the risk of administrative errors. At the end of the AFIS process, the system automatically generates AFIS reports containing the opinions and charts of each independent examiner. The AFIS workflow of the NPN enables the automatic incorporation of personal and case information into the report. The report includes charts generated by the system and examiners involved in the analysis. To ensure accuracy and accountability, the examiners co-signed the printed reports. The last stage of the process is executed by the lead specialist and involves conducting Quality Assurance (QA) and Quality Control (QC) checks to ensure that the standard operating procedure is meticulously followed, and that the information contained in the printed report is entirely accurate and error-free.

Supported by the software, the initial charting is first performed manually by the examiner prior to the generation of the final court report. This process involves searching for corresponding features in the mark and reference print based on the appearance, location, position, direction of the feature, and other surrounding information. If a feature is reproducible and corresponds to a feature in the print, a line is drawn, and the features are assigned the same number to indicate correspondence. This process is repeated until the minimum standard set by the NPN policy is reached, and no unexplainable differences are observed. Not all features are charted and once the minimum standard set by the NPN policy is reached, charting may be stopped. It is important to note that NPN follows a numerical reporting standard. Upon completion of the charting process, a court chart is automatically generated by the AFIS. Once the examiner completes charting, the chart is automatically added to the PDF report. Full ACE reports are accompanied by a court chart in the case file.

**Fig. 9:**
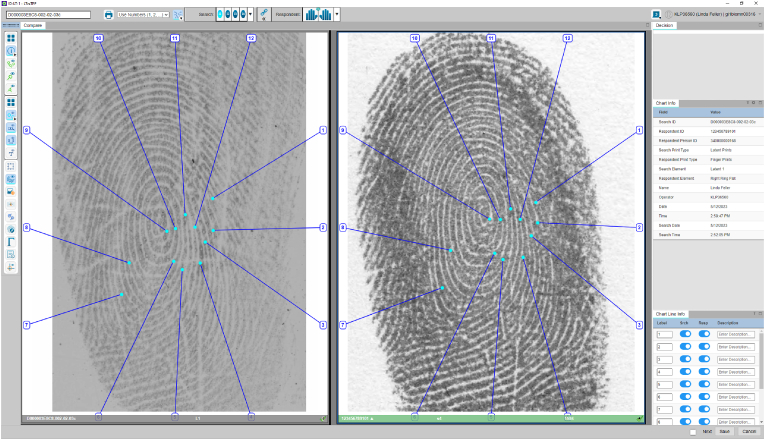
Screenshot of the NPN AFIS interface displaying the court chart for demonstration purposes. The examiner begins by pairing the associated minutiae, and each line in the chart corresponds to a specific feature marked by the examiner, with numbers at the end of each line indicating the associated features. For example, a feature marked ‘1′ in the fingermark is linked to the feature marked ‘1′ in the reference print, and so on.

Based on the original analysis paired with the observed natural distortion, it is not uncommon to observe some differences between the features in the mark and print. For this reason, a degree of explainable difference is acceptable, especially if the original feature has been marked yellow or red during the GYR markup, indicating that a feature exists, but a lower confidence is applied during comparison to account for distortion. This also holds true for features that exhibit connective ambiguity and can be classified as either ridge ending or bifurcation, and depending on the deposition pressure may appear to be one ridge count out. An example of an explainable difference can be observed in Fig. 9. In this case, feature 1 is clearer on the mark, but is observed in a distorted area on the reference print. The combination of distortion and connective ambiguity may give the impression of being one ridge count off.

### 6.9 Consensus in practice

The NPN AFIS workflow is notable for successfully implementing a consensus approach, which involves a minimum of two examiners reaching a general agreement, and a third, fourth, and fifth examiner being initiated if a difference of opinion is reached. The system “loops” or feeds every mark back into the system a maximum of three times, meaning the search, including encoding, launching, comparing, and evaluating the candidate list to determine a positive or negative comparison decision, is performed by multiple examiners independently of one another. The examiners analysing the mark are unaware of who the other examiner and what the result of the previous examination is. This approach mirrors the “wisdom of the crowd " approach, which involves pooling judgments to prevent errors in high-stakes decision-making domains such as medicine and aviation [52].

## 7 Other considerations for establishing best practice approaches

AFIS managers must consider several factors beyond the workflow process when establishing best-practice approaches in an operational environment. Some of these factors include international standards, AFIS examiners, and purchasing an AFIS.

### 7.1 International standards (ISO)

International standards emphasise the need for laboratories to plan and implement actions that address risks and opportunities and outline the requirements for competence, impartiality, freedom from bias, and consistent operation of laboratories. The ISO 17025 standard also outlines five requirements for processes, procedures, documented information, and organisational responsibilities. These requirements cover various aspects of laboratory operations, including the general, structural, resource, process, and management system requirements. In the context of vendors that supply agencies with AFIS, it is essential to pay attention to the process requirements outlined in ISO 17025.

One critical process requirement is the need for the laboratory to perform a procedure for reviewing requests, tenders, and contracts. This procedure is an essential component of an operational environment. The laboratory should also inform the vendor when the requested method is inappropriate or outdated. Therefore, to effectively implement and validate updated processes, operational managers involved in tender negotiations must keep up-to-date with scientific and technological advancements. This ensures that the methods and examiners remain current, competent, and valid.

### 7.2 The AFIS examiner

Factors such as talent, training, experience, and mental state can significantly influence their work. Therefore, it is important to develop human strategies beyond the workflow process. This should include careful considerations of recruitment, training, proficiency, competency, and ongoing education, as well as the separation of tasks and responsibilities based on levels of expertise. Additionally, annual eye testing should be conducted to ensure that the examiner's visual capabilities are up to par [18].

### 7.3 Purchasing an AFIS

In operational environments, purchasing an AFIS is usually accomplished through an upgrade of the existing system or through the procurement of a completely new AFIS. There are differences in the accuracy and performance of the algorithm among AFIS vendors, making it important to understand the algorithm being purchased. In addition to the accuracy of the algorithm, there are many other performance requirements such as cost, speed, and maintainability, which ensure that the system is effective and efficient.

Purchasing an AFIS in Europe is a complex process that typically follows the EU tender rules. An open tender process may be used, and a formula is often used to assess all requirements and determine a winner for the bid. This involves specifying a multitude of demands and requirements, which can sometimes rule out potentially interesting vendors who may not be able to fully comply with all demands. Therefore, it is important to be careful when specifying these demands. In contrast, wishes about the functionality of AFIS (i.e., what you would like on your AFIS and what the AFIS vendor can provide) are not mandatory (i.e., what you must have on AFIS regardless of vendor) and can be more open to discussion, both technically and economically, during the tender at a high level and once the tender has been awarded to a specific vendor in detail.

Furthermore, the maintainability of a system is essential to ensure that it continues to function effectively over time. A well-maintained system reduces the risk of errors, prevents downtime, and ensures the continuity of operations. Additionally, other factors such as data security, privacy, and compliance with regulations should be considered to ensure that the AFIS system meets the necessary standards and requirements. It is essential to choose the most effective AFIS solution suitable for the intended purpose, and finding the most accurate technology is an important aspect.

However, accuracy may come at a price, not only in terms of economics, but also in terms of maintenance and user-friendliness. Organising a benchmark (see 2.2.1) to evaluate and compare different AFIS solutions requires specialised expertise and introduces potential risks in terms of disputes with vendors who do not agree with the benchmark's outcome. When selecting an AFIS, it is necessary to evaluate its overall performance based on a combination of different factors such as accuracy, speed, cost, scalability, reliability, wishes, and ease of maintenance. For instance, although accuracy is critical in identifying potential suspects, the cost of the system is a significant factor in determining its feasibility and sustainability over the long term. Speed is also a factor to be considered when processing biometric data, particularly in high-pressure forensic operational settings. However, there can be a trade-off between speed and accuracy.

## 8 Concluding remarks

The AFIS is a widely used forensic operational tool used to verify the identity of individuals and to search and associate crime-related marks against the reference database. Although automated biometric systems have positively contributed to public safety efforts, traditional systems may not be best equipped to support forensic processes in best practice approaches. To improve AFIS performance, it is essential to manage a combination of organisational, system, and human factors.

In this paper we have emphasised the importance of understanding the limitations of the examiner and AFIS technology. Additionally, we benchmarked the AFIS of the NPN, which has implemented strategies in its AFIS that reflect aspects of recommended best practice, such as context management, separation of tasks, and documentation strategies, to improve the accuracy and performance of the examiner and the system. NPN have implemented a consensus approach in their AFIS workflow in which examiners process the same mark independently and blindly.

In conclusion, we encourage AFIS managers and end users to study other agencies and adopt best practices to promote greater harmonisation and improve performance while minimising risks. International standards, forensic standards, guidelines, and recommended best practices should guide decisions regarding AFIS upgrade. It is critical to prioritise the accuracy and performance of both the examiner and the AFIS in the interest of public safety when purchasing and upgrading AFIS systems.

## 9 Declaration of AI-assisted technologies in the writing process

Statement: During the preparation of this paper, the author(s) used OpenAI (ChatGPT) to improve readability and language, following major revisions. After using this tool/service, the author(s) reviewed and edited the content as needed and takes(s) full responsibility for the content of the publication. Furthermore, all the contents are original and written by the authors. The tool was solely employed during the major revision phase to assess readability and language, if necessary, and not to generate any content.

## Declaration of competing interest

The authors declare the following financial interests/personal relationships which may be considered as potential competing interests:Dear Editor, John Riemen is a Member of the IDEMIA Justice and Public Safety Executive Users Board, where he represents the National Police of the Netherlands (NPN) in his function as Chief Inspector - Lead Biometric Specialist, and Manager of the National Criminal Automated Biometric Identification System (ABIS). The authors would like to emphasise that this affiliation is not in conflict with the objectives of this manuscript. It is not a marketing paper for a commercial company. The audience are AFIS managers and end-users. The primary objective was to benchmark the Dutch operational AFIS strategies, and to share AFIS capabilities which align with scientific best practice with the forensic fingerprint community. The authors do not gain any advantage, financial or other. For your consideration, Caroline Gibb and John Riemen.
